# A Smartphone-Based Approach for Triage of Human Papillomavirus-Positive Sub-Saharan African Women: A Prospective Study

**DOI:** 10.2196/mhealth.6697

**Published:** 2017-05-29

**Authors:** Esther Urner, Martine Delavy, Rosa Catarino, Manuela Viviano, Ulrike Meyer-Hamme, Anne-Caroline Benski, Jeromine Jinoro, Josea Lea Heriniainasolo, Manuela Undurraga, Hugo De Vuyst, Christophe Combescure, Pierre Vassilakos, Patrick Petignat

**Affiliations:** ^1^ Gynecology Division Department of Gynecology and Obstetrics Geneva University Hospitals Geneva Switzerland; ^2^ Laboratory Division Saint Damien Healthcare Centre Ambanja Madagascar; ^3^ Gynecology Division Department of Gynecology and Obstetrics Saint Damien Healthcare Centre Ambanja Madagascar; ^4^ International Agency for Research on Cancer World Health Organization Lyon France; ^5^ Research Center Geneva University Hospitals Geneva Switzerland; ^6^ Geneva Foundation for Medical Education and Research Geneva Switzerland

**Keywords:** cervical cancer, squamous intraepithelial lesions of the cervix, HPV, acetic acid, lugol’s iodine, smartphone, mobile phone

## Abstract

**Background:**

Sub-Saharan African countries are marked by a high incidence of cervical cancer. Madagascar ranks 11th among the countries with the highest cervical cancer incidence worldwide.

**Objective:**

The aim of the study was to evaluate the performances of digital smartphone-based visual inspection with acetic acid (D-VIA) and Lugol’s iodine (D-VILI) for diagnosing cervical precancer and cancer.

**Methods:**

Human papillomavirus (HPV)-positive women recruited through a cervical screening campaign had D-VIA and D-VILI examinations with endocervical curettage (ECC) and cervical biopsy. Three images were captured for each woman (native, D-VIA, D-VILI) using a smartphone camera. The images were randomly coded and distributed on 2 online databases (Google Forms). The D-VIA form included native and D-VIA images, and the D-VILI form included native and D-VILI images. Pathological cases were defined as cervical intraepithelial neoplasia grade 2 or worse (CIN2+). Physicians rated the images as non-pathological or pathological. Using the ECC and cervical biopsy results as references, the sensitivity and specificity of D-VIA and D-VILI examinations for each and all physicians were calculated.

**Results:**

Altogether, 15 clinicians assessed 240 images. Sensitivity was higher for the D-VIA interpretations (94.1%; 95% CI 81.6-98.3) than for the D-VILI interpretations (78.8%; 95% CI 54.1-92.1; *P*=.009). In contrast, the specificity was higher for the D-VILI interpretations (56.4%; 95% CI 38.3-72.9) than for the D-VIA interpretations (50.4%; 95% CI 35.9-64.8; *P*=.005).

**Conclusion:**

Smartphone-based image for triage of HPV-positive women is more accurate for detecting CIN2+ lesions with D-VIA than D-VILI, although with a small loss of specificity.

## Introduction

Sub-Saharan African countries are marked by a high incidence of cervical cancer [1]. Madagascar, in particular, counts about 3194 new cervical cancer cases every year and ranks 11th among the countries with the highest cervical cancer incidence worldwide [1].

Because of the long interval between the initial human papilloma virus (HPV) infection and the development of cervical cancer, screening has proven to be largely effective in reducing the incidence of cervical cancer in industrialized countries [2]. In low- and medium-income countries (LMICs), however, the lack of expertise and infrastructure makes screening programs difficult to implement. To overcome this difficulty, one of the options recommended by the World Health Organization (WHO) includes HPV testing followed by visual inspection of the cervix with acetic acid (VIA) as a triage test [3]. A meta-analysis, however, has shown that visual inspection with Lugol’s iodine (VILI) may perform better than VIA [4].

Given the concerns about the suboptimal sensitivity of VIA/VILI tests and the lack of a quality assurance system, the use of digital VIA (D-VIA) and D-VILI is a promising choice for LMICs. Smartphone cameras are easy to use, have excellent image quality, and by sending the images to other on-site and off-site experts, they offer the possibility to obtain real-time feedback.

A previous study conducted in Madagascar has shown that off-site detection of cervical lesions based on the evaluation of smartphone photographs was more reliable than on-site diagnosis alone [5]. The aim of this study was to assess the clinical performance of D-VIA and D-VILI examinations for diagnosing cervical lesions in LMICs.

## Methods

The cervical cancer screening campaign took place in the Saint Damien Healthcare Centre in Ambanja, Madagascar and in five dispensaries in the surrounding rural areas between February and October 2015. As a means of primary screening, women were asked to collect a vaginal self-sample with a sterile swab (ESwab, Copan, Brescia, Italy). The samples were then analysed in Ambanja by a point-of-care HPV assay (GeneXpert IV; Cepheid, Sunnyvale, CA, USA). Women who tested positive for high-risk HPV genotypes were invited to the Saint Damien Healthcare Centre to undergo a gynecological examination. Women were included in the study if they were aged between 30 to 69 years, HPV-positive for high-risk HPV genotypes, able to understand study procedures, and voluntarily participated by signing the informed consent form. Exclusion criteria included a previous hysterectomy, conditions that could interfere with visualization of the cervix, pregnancy over 20 weeks, or that they could not comply with study protocol. This cross-sectional analysis included data from HPV-positive women with a disease prevalence corresponding to that of real-life conditions (10–15% cervical intraepithelial neoplasia grade 2 or worse [CIN2+]).

All HPV-positive patients underwent digital VIA/VILI examinations, which were carried out on the same day or a few weeks following self-HPV testing. Three images per examination were captured using a smartphone (Samsung Galaxy S4 or S5, Seoul, South Korea). The first image corresponded to the native cervix. The second was captured one minute after application of a 5% acetic acid solution and the third following application of Lugol’s iodine. Cervical (Papanicolaou) smears, endocervical curettage and an average of 2 biopsies were performed on all patients. If no lesion was identified, a random cervical biopsy was performed at the 6 o’clock site. Patients for whom a pathological cervical lesion (CIN2+) was suspected based on the immediate evaluation by the on-site expert were treated with cold coagulation or large electrosurgical excision procedure (LEEP) on the same day. A hysterectomy was scheduled for the following days when invasive cervical cancer was suspected, if operable. Women who were not promptly treated on site but who were later diagnosed histologically with cervical dysplasia were called again to the Saint Damien Healthcare Centre for further management.

The GeneXpert device was used on site for HPV DNA detection. Using a polymerase chain reaction method, this test detects 14 types of high-risk HPV. The results are divided into three sections: HPV 16, HPV 18/45, and 11 other high-risk HPV types (31, 33, 35, 39, 51, 52, 56, 58, 59, 66, and 68). Endocervical samples were collected using an endocervical brush. A cervical biopsy forceps was used for histological specimen collection. Both samples were fixed in liquid formalin. The reference standard for the disease was the histological evaluation, which was performed in Geneva, Switzerland. The results were interpreted according to the WHO 2014 classification as Low-grade squamous intraepithelial lesions (LSIL) and High-grade squamous intraepithelial lesions (HSIL) and sub-classified as grade 1, 2, or 3 CIN.

Throughout the pelvic examination, photographs were obtained at a distance of 10-15 cm of the cervix, with 3.3-3.8× optical zoom and in flash mode. The two smartphones, Samsung Galaxy S4 and S5, were chosen for their high-quality cameras (13 and 16 megapixels, respectively, both with auto-focus and flash functions). These devices allow highly precise and detailed visualization of the cervix after zooming and focusing in on the target. To improve the stability and quality of the images, the smartphone was fixed to the ground with a tripod and a support.

**Figure 1:**
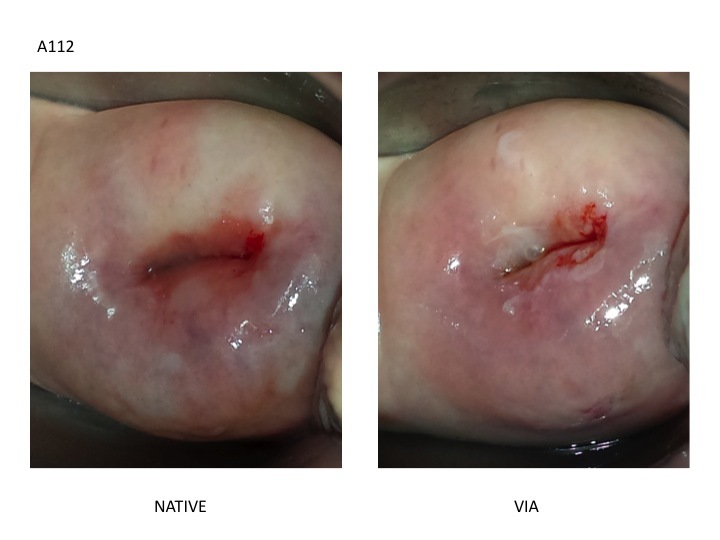
Digital visual inspection using acetic acid (D-VIA). Left. Native cervix. Right. Cervix after acetic acid application.

**Figure 2:**
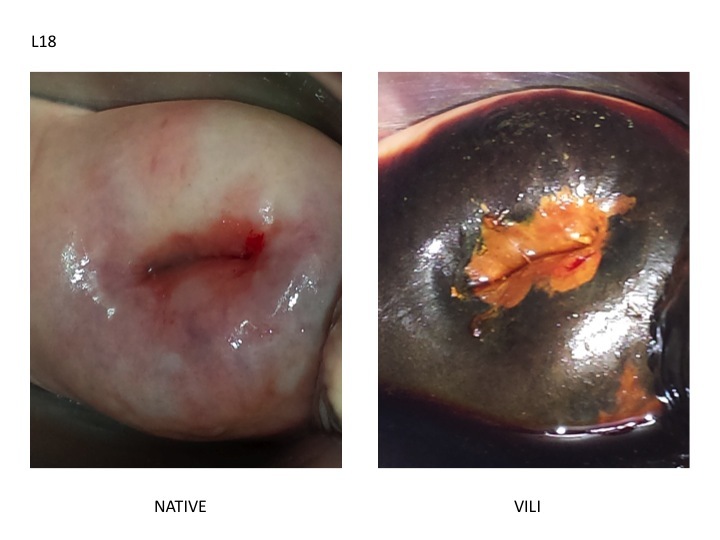
Digital visual inspection using Lugol’s iodine (D-VILI). Left. Native cervix. Right. Cervix after Lugol’s iodine application.

The images were uploaded onto two online databases (Google forms). One form included the native and D-VIA images. The second included the native and D-VILI images. The images were randomly coded and distributed on the two databases so that the D-VIA and D-VILI images of each patient could not be linked to one another. Overall, 15 physicians with different clinical backgrounds and experience, blind to the histological and HPV genotypes, were asked to determine (for each patient in each of the two Google forms) whether the images of the cervix were non-pathological (<CIN2) or pathological (CIN2+) using IARC Reference Chart for Visual Inspection with acetic acid (VIA) and Lugol’s iodine (VILI). Figures 1 and 2 represent a CIN3 case presented on the D-VIA and D-VILI forms, respectively. The physicians also completed a questionnaire addressing their own background and experience in colposcopy (Table 1). The examining physicians were labeled as “experts” if the number of colposcopies performed in their lifetime was greater than 300 and if they had at least ten years of experience in clinical practice. Out of the 15 physicians, 5 fulfilled these criteria and were labeled as “experts.” The remaining 10 physicians had performed less than 300 colposcopies, had less than 10 years of experience in clinical practice, and were labeled as “novices.”

The sensitivity and specificity for the detection of CIN2+ were calculated for the D-VIA and D-VILI interpretations of each rater. The correlation coefficients between screener-specific sensitivities and specificities were assessed for both D-VIA and D-VILI (Spearman coefficient of correlation). To assess the global sensitivity and specificity (for all readers), we used logistic regression models with mixed effects to account for repeated measures: a random intercept was introduced into the model, and the random effects for patients and readers were crossed. Models were applied to patients with a positive biopsy to assess sensitivity and to patients with a negative biopsy to assess specificity. Additional logistic regression models with mixed effects were used to test a difference in sensitivity or specificity between expert screeners and novice screeners and to test a difference between D-VIA and D-VILI.

All statistical tests were two-sided, with a type 1 error of 0.05. Statistical analyses were performed with R Core Team 2015 software (R Foundation for Statistical Computing, Vienna, Austria) and Stata13 IC software (StataCorp, College Station, TX, USA).

The study was approved by the Malagasy National Commission for the Ethics of Science and Technology and the Ethical Cantonal Board of Geneva, Switzerland (CER: 14–071). The trial has been registered on clinicaltrials.gov (NCT02693379).

## Results

Among the 1041 participants screened for HPV, 231 were HPV-positive (22.2%) and 584 HPV-negative (77.8%). Among HPV-positive women, 187 underwent digital VIA/VILI testing in the Saint Damien Health Care Centre. A total of 561 images were captured during the examination. A total of 120 out of 187 (64.2%) sets of photographs of women who underwent digital VIA/VILI were considered of good quality and were included in the study. Images were excluded because of insufficient focus or inadequate magnification. The resulting disease prevalence in our study corresponds to that of an unscreened asymptomatic population (10-15%) [6].

The overall distribution of cervical disease among the 120 patients was 14 (11.6%) pathological cases (CIN2+) that included 4 (3.3%) cases of invasive cancer. There were 7 (5.8%) cases of CIN3, 3 (2.5%) cases of CIN2, and 4 (3.3%) cases of CIN1. In all, 102 (85.0%) cases were negative for any cervical lesion. CIN1 lesions were considered and managed as negative cases. Overall, 14 out of 65 (21.5%) CIN2+ cases were promptly treated with thermocoagulation or conisation, whereas 51 out of 64 (79.7%) cases were overtreated.

The raters’ mean age was 39.7 years (SD 8.3). Their mean number of years of experience in clinical practice was 11.0 years (SD 6.5). Table 1 reports the sociodemographic characteristics of the 15 raters.

**Table 1:** Raters’ sociodemographic characteristics.

Variable		n (%)
**Total**		15
**Age (years), mean (SD)**		39.7 (SD 8.3)
**Nationality**		
	Swiss	7 (46.7)
	Other	8 (53.3)
**Profession**		
	Medical Doctor	14 (93.3)
	Other	1 (6.7)
**Years of experience in clinical practice, mean (SD)**		11.0 (SD 6.5)
**Number of colposcopies during lifetime**		
	0	1 (6.7)
	<50	6 (40.0)
	51–300	3 (20.0)
	>300	5 (33.3)
**Number of colposcopies per year**		
	0	4 (26.7)
	<30	8 (53.3)
	>60	3 (20.0)

The diagnostic performance of D-VIA and D-VILI varied broadly among raters: the overall sensitivity was 81.6-98.3% for D-VIA and 54.1-92.1% for D-VILI. The specificity was 35.9-64.8% for D-VIA and 38.3-72.9% for D-VILI. For both techniques, the specificity decreased as the sensitivity increased (ρ=−0.59 for D-VIA and ρ=−0.80 for D-VILI). The overall sensitivity was 94.1% (95% CI 81.6-98.3) for D-VIA and 78.8% (95% CI 54.1-92.1) for D-VILI (*P*=.009). The overall specificity was 50.4% (95% CI 35.9-64.8) for D-VIA and 56.4% (95% CI 38.3-72.9) for D-VILI (*P*=.005).

**Figure 3:**
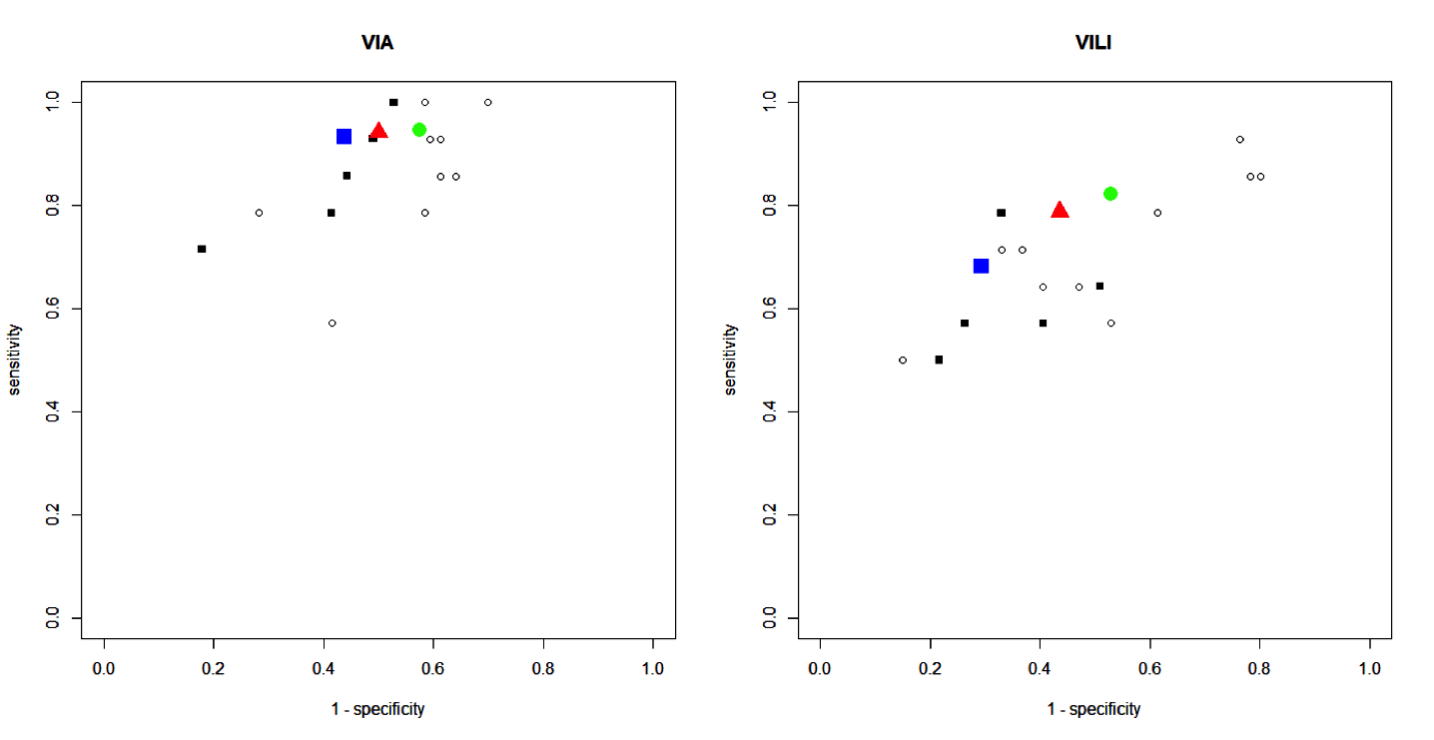
Sensitivity and specificity of digital visual inspection with acetic acid (D-VIA) and Lugol’s iodine (D-VILI) for all raters (red triangle), experts (blue square), and novices (green circle).

The specificity was higher for experts than novices, but the difference was statistically significant only for D-VIA (*P*=.04). No statistically significant differences between experts and novices were found concerning sensitivity. Among the expert raters, D-VIA had higher sensitivity than D-VILI (*P*=.01) and lower specificity (*P*=.02). In novice raters, the trend of the findings was the same, but the difference in specificities and sensitivities were not statistically significant (*P*=.07 and *P*=.05, respectively). Table 2 and Figure 3 report the sensitivity, specificity, and *P* values for D-VIA and D-VILI among all raters, experts, and novices.

**Table 2:** Sensitivity, specificity, and *P* value for D-VIA and D-VILI among all raters, experts, and novices

Parameters		D-VIA^a^ Overall (range)	D-VILI^b^ Overall (range)	*P*^c^
Sensitivity				
	All raters (n=15)	94.1% (81.6-98.3)	78.8% (54.1-92.1)	.009
	Experts (n=5)	93.3% (67.3-99.0)	68.5% (37.8-88.6)	.01
	Novices (n=10)	94.6% (78.8-98.8)	82.3% (58.3-94.0)	.05
*P* (experts vs novices)		.86	.12	
Specificity				
	All raters (n=15)	50.4% (35.9-64.8)	56.4% (38.3-72.9)	.005
	Experts (n=5)	56.2% (44.5-81.4)	70.6% (57.0-81.3)	.02
	Novices (n=10)	42.5% (28.5-57.9)	47.0% (26.2-68.9)	.07
*P* (experts vs novices)		.04	.08	

^a^D-VIA: digital visual inspection with acetic acid.

^b^D-VILI: digital visual inspection with Lugol’s iodine.

^c^D-VIA vs D-VILI: vExperts vs. Clinical PDntion either “e either” re prefrred.

## Discussion

### Principal Findings

Our study shows that the use of digital images for triaging HPV-positive women allows detection of most precancerous and cancerous lesions. When considering all raters, D-VIA showed a significantly higher sensitivity than D-VILI, whereas D-VILI was associated with significantly higher specificity. Furthermore, the specificity of D-VIA was higher among the more experienced screeners than among the less experienced ones. Although we did not compare these digital inspection methods with naked-eye techniques, this last finding could relate to the fact that bare-eye VILI is relatively easier to interpret than VIA, whose validity relies more on the experience and training level of the health worker [4].

As the overall sensitivity was higher for D-VIA, this study supports the superiority of D-VIA to D-VILI for CIN2+ detection. This result is partly in line with the WHO policy, according to which a primary visual approach should be VIA-based [3]. Furthermore, with a sensitivity of 94.1% and specificity of 50.4% for the detection of CIN2+, D-VIA showed a performance comparable with the one found in a study evaluating the performance of a low-cost magnifying device, the Magnivisualiser, where VIA had a sensitivity of 88.3% and a specificity of 55.8% [6].

Although VIA is currently recommended as a single screening tool, VILI is used to aid health workers in making the diagnosis because of its higher specificity and facility to interpret, especially for less-experienced health care providers. The cost and availability of Lugol’s iodine, however, may potentially be an obstacle to the use of VILI in LMICs [7], resorting to the use of VIA alone in some regions.

In comparison to a recent meta-analysis on cervical cancer screening using naked-eye inspection methods which states that VILI seems to be the most sensitive test to use in the African continent, our study shows that D-VIA is more sensitive for detecting cervical lesions than D-VILI (94.1% vs 78.8%) [8]. The higher sensitivity obtained with D-VIA compared with D-VILI could be explained by the fact that, in this context, the raters were able to examine the images for a longer period of time and to compare the native photographs consecutively with those with D-VIA. This is not possible with the bare-eye approach because, once the application of Lugol’s iodine is completed, the cervix appears as brown or black and the native and acetic acid appearance cannot be seen anymore. In addition, because the images were exclusively of HPV-positive women, the raters may have been inclined to give them a positive score in anticipation of a higher disease proportion in this group than in the general screening population.

In a low-income setting, such as that of Madagascar, cervical cancer screening tools that are commonly used in industrialized countries such as Pap smear and colposcopy, coupled with the continuous training of health care providers, are unlikely to be systematically available. The introduction of a smartphone-based approach for cervical cancer screening in such settings would allow to overcome some of the barriers to the implementation of screening programs in developing countries. The capture of cervical images with the smartphone camera allows the user to look back at either the native, post-VIA, or post-VILI cervix and to magnify the pictures in order to see them more closely before deciding whether or not to treat. In addition, the automatic saving of digital images on the smartphone allows the on-site, often less experienced and less qualified healthcare worker, to seek advice from long-distance off-site specialists [9].

Moreover, the use of automated phone applications is on the rise and might improve and facilitate CC screening in LMICs by providing a system to classify the images and to guide health workers through their decision-making algorithm [10]. Such mobile health tools are either free of charge or come at a very low price and can be easily installed on a smartphone without requiring any additional equipment. Their low cost and practicality distinguish them from other mobile colposcopy systems, such as the MobileODT (EVA system, enhanced visual assessment; Tel Aviv, Israel) for which the digital images’ increased sharpness comes at the cost of a far more expensive and elaborate type of equipment. These aspects make the use of images taken by mobile phone a promising option for cervical cancer screening in low-income countries. Whether D-VIA and D-VILI should be used as a triage test for HPV-positive women or as a stand-alone screening tool is a question that yet remains unanswered. Further prospective studies are needed in order to assess the performance of D-VIA and D-VILI as a single, co-testing or triage screening tool.

### Strengths and Limitations

The strengths of our study were the large number of consecutive cases included in a “triage context” and the fact that all of them underwent ECC and cervical biopsy, which served as the gold standard. In addition, patient recruitment and data collection took place in a real-world setting, which entails several consecutive, unselected cases.

Limitations that need to be addressed are the fact that D-VIA and D-VILI images of each patient were assessed separately rather than consecutively. Another limitation is the fact that most of our raters were medical doctors, which does not allow us to validate our results for all healthcare workers, such as nurses and midwives. The technical difficulties encountered in obtaining high quality images also did not allow us to include pictures of all the patients in the analysis. Additional practice is necessary in order to improve the quality of the images before transferring the smartphone use on to a real-life setting.

### Conclusions

In conclusion, our study emphasizes the higher sensitivity of D-VIA when compared to D-VILI for detecting precancerous lesions, although at the cost of slightly lower specificity. Further research should focus on comparing the performance of these two digital screening techniques with naked-eye methods and on assessing their feasibility in a low-resource context.

## Abbreviations

CIN1: cervical intraepithelial neoplasia grade 1
CIN2: cervical intraepithelial neoplasia grade 2
CIN2+: cervical intraepithelial neoplasia grade 2 or worse
CIN3: cervical intraepithelial neoplasia grade 3
D-VIA: digital smartphone-based visual inspection of the cervix with acetic acid
D-VILI: digital smartphone-based visual inspection of the cervix with Lugol’s iodine
ECC: endocervical curettage
HPV: human papillomavirus
LMICs: low- and medium-income countries
VIA: visual inspection of the cervix with acetic acid
VILI: visual inspection of the cervix with Lugol’s iodine
WHO: world health organization
